# Topographic correlation and asymmetry analysis of ganglion cell layer thinning and the retinal nerve fiber layer with localized visual field defects

**DOI:** 10.1371/journal.pone.0222347

**Published:** 2019-09-11

**Authors:** Alfonso Casado, Andrea Cerveró, Alicia López-de-Eguileta, Raúl Fernández, Soraya Fonseca, Juan Carlos González, Gema Pacheco, Elena Gándara, Miguel Á. Gordo-Vega

**Affiliations:** Department of Ophthalmology, Hospital Universitario Marqués de Valdecilla-IDIVAL, Santander, Spain; University of Miami Bascom Palmer Eye Institute, UNITED STATES

## Abstract

**Purpose:**

To evaluate the accuracy of the measurement of the ganglion cell layer (GCL) of the posterior pole analysis (PPA) software of the Spectralis spectral-domain (SD) optical coherence tomography (OCT) device (Heidelberg Engineering, Inc., Heidelberg, Germany), the asymmetry of paired GCL sectors, the total retinal thickness asymmetry (RTA), and the peripapillary retinal nerve fiber layer (pRNFL) test to discriminate between healthy, early and advanced glaucoma eyes.

**Methods:**

Three hundred eighteen eyes of 161 individuals with reliable visual fields (VF) were enrolled in this study. All participants were examined using the standard posterior pole and the pRNFL protocols of the Spectralis OCT device. VF impairment was graded in hemifields, and the GCL sectors were correlated with this damage. Thicknesses of each GCL, the GCL map deviation asymmetry and the pRNFL were compared between control and glaucomatous eyes. The area under the receiver operating characteristic curve (AUC) of these analyses was assessed.

**Results:**

Fourteen of the 16 sectors of the GCL and pRNFL were significantly thinner in eyes with glaucoma than in control eyes (p<0.006). Similarly, the GCL map deviation showed a significant difference between these eyes and both the control eyes as well as the eyes with early glaucoma (p = 0.001 and p = 0.039, respectively). The highest values of AUC to diagnose both early and advanced glaucoma corresponded to the average pRNFL analysis and the GCL map deviation (AUC>0.823, p<0.040 and AUC>0.708, p<0.188, respectively).

**Conclusions:**

Although 16 central sectors of the GCL observed with PPA showed good correlation with VF damage, the pRNFL and the GCL map deviation were more effective for discrimination of glaucomatous damage.

## Introduction

Glaucoma is the principal aetiology of irreversible blindness worldwide [1]. Glaucoma is an optic neuropathy that causes optic nerve head damage and retinal ganglion cells impairment [2,3]. Using optical coherence tomography (OCT), we are able to analyse both structures by measuring the peripapillary retinal nerve fiber layer (pRNFL) as well as the ganglion cell layer (GCL) [4]. Thus, both measurements are used for clinical glaucoma assessments [5–7]. The pRNFL thickness analysis is currently the most commonly used OCT parameter [7]. However, the GCL has recently become a target for early structural glaucoma detection, as progressive GCL thinning might be an essential pathology of glaucoma [8–11]. Previous reports have shown the utility of such measurement for glaucoma diagnosis using various parameters, such as ganglion cell-inner plexiform layer (GCIPL) [12, 13]. These studies reported that the glaucoma- diagnostic performance of the GCIPL parameters was similar to that of the pRNFL parameters that have been widely applied for the diagnosis of glaucoma. In fact, other reports showed that the GCL thickness decreased earlier than the pRNFL in early glaucoma [14, 15].

The correlation between visual field (VF) damage and structural changes measured with OCT has always been a challenging task, as VF uses logarithmic data differently from OCT analyses. However, several studies have tried to correlate both tests to improve our ability to detect the presence and progression of glaucomatous damage. The results of these studies have proven there is a high interdependence between the global VF sensitivity and the pRNFL/GCL thicknesses [16–18]. However, a good correlation has not yet been proposed [19]. This correlation might be assisted by a recent update of the Spectralis OCT or the Posterior Pole Analysis (PPA) (Heidelberg Engineering, Heidelberg, Germany), which automatically delineates 61 line scans to provide 64 sectors of the measured GCL [20]. This measurement allows us to obtain a large amount of information on the GCL condition that might be used for correlation with VF, as well as to test the asymmetry between hemispheres. In fact, previous reports have stated that the analysis assessing the difference in macular total retinal thickness between retinal hemispheres might be an indicator of glaucomatous damage [21,22]. However, the asymmetry of the GCL using PPA was not previously tested and, regarding GCIPL results using OCT Cirrus [12–15], it may be useful for OCT Spectralis as well.

Thus, the aims of the current study were to assess the efficacy of different GCL asymmetry analyses (GCL map deviation and 16 different sectors of GCL). In addition, we sought to perform a comparison with PPA automatic asymmetry analysis and the pRNFL to diagnose early and advanced glaucomatous impairment, as well to correlate different numeric patterns of GCL thinning with VF damage.

## Methods

This prospective, cross-sectional, observational study complied with the tenets of the Declaration of Helsinki and was approved by the Ethical Committee of University Hospital Marqués de Valdecilla (UHMV). All participants were recruited from the ophthalmology department of Valdecilla Hospital from June 2016 to December 2018. Written consent forms were distributed to all the participants before the examinations.

All subjects were required to have a refractive error less than -6.0 diopters of sphere or 3 diopters of cylinder, as well as less than 26 mm of axial length. Since retinal layer’s thicknesses are related with glaucoma and other various retinal pathologies, we excluded all patients with other ocular diseases (for example, diabetic retinopathy, macular degeneration, optic neuritis), clinically relevant opacities of the optic media and low- quality images due to unstable fixation, or severe cataract (patients with mild to moderate cataract could be enrolled in the study, but only high-quality images were included). Three hundred eighteen eyes from 161 patients examined in the glaucoma section were enrolled in the study (4 eyes were excluded due to low reliability of the VF).

### Clinical assessment

All subjects underwent a thorough ophthalmic examination on the day of OCT imaging, including best-corrected visual acuity, refraction, intraocular pressure (IOP) measurement with GAT, slit lamp examination and fundus examination. The refractive error was recorded using an auto refractometer Canon RK-F1 (Canon USA Inc., Lake Success, NY, USA). Axial length was measured by Lenstar LS 900 (Haag Streit AG, Koeniz, Switzerland).

### Optical coherence tomography procedure

#### Methodology for measurement of the GCL

**Fig 1:**
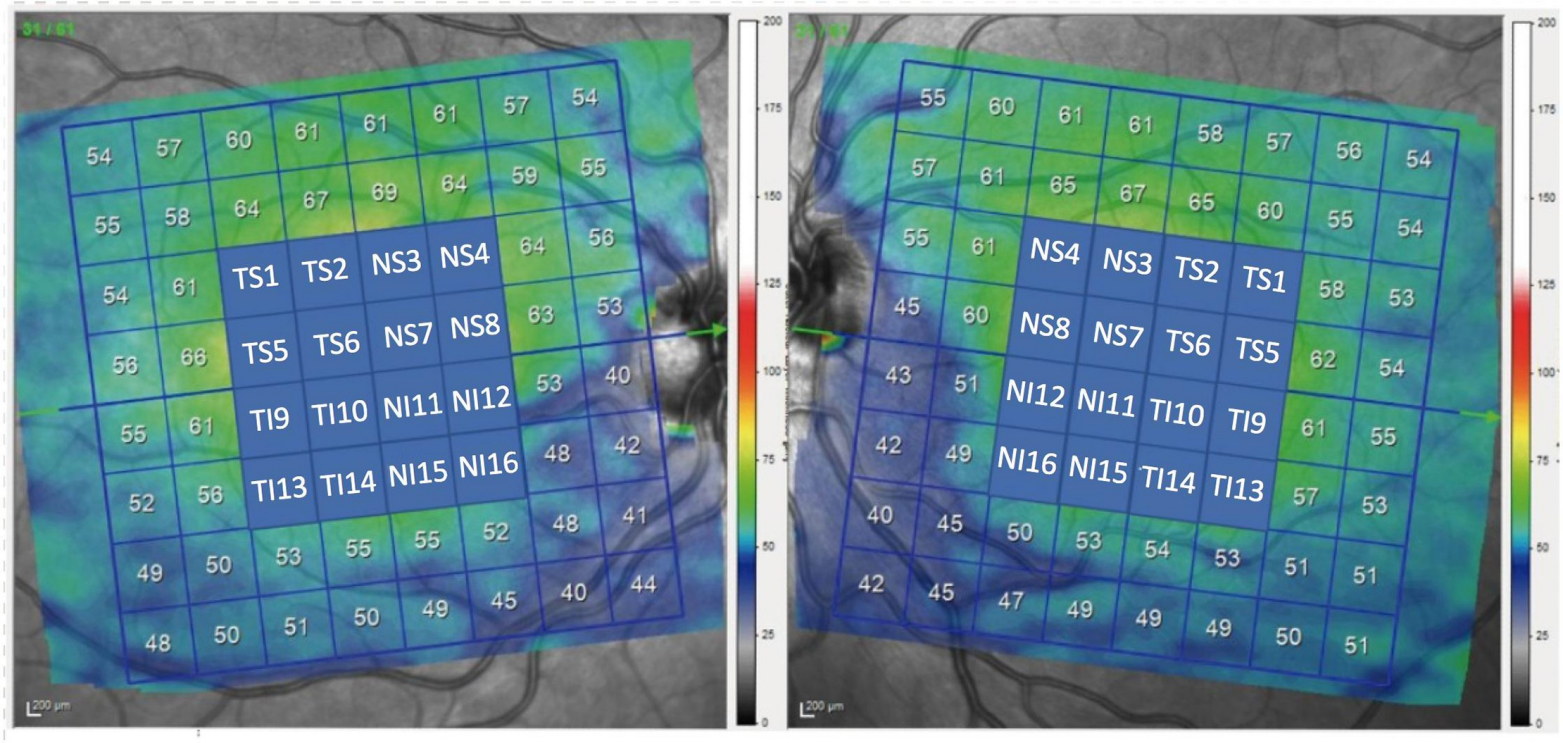
OCT scan of the macula with the posterior pole analysis (PPA), showing the 16 sectors numbered from 1 to 16. Nasal sectors included numbered NS3, NS4, NS7, NS8, NI11, NI12, NI15 and NI16 sectors; temporal sectors included TS1, TS2, TS5, TS6, TI9, TI10, TI13 and TI14; superior sectors were from 1–8, whereas inferior sectors were from 9–16.

**Fig 2:**
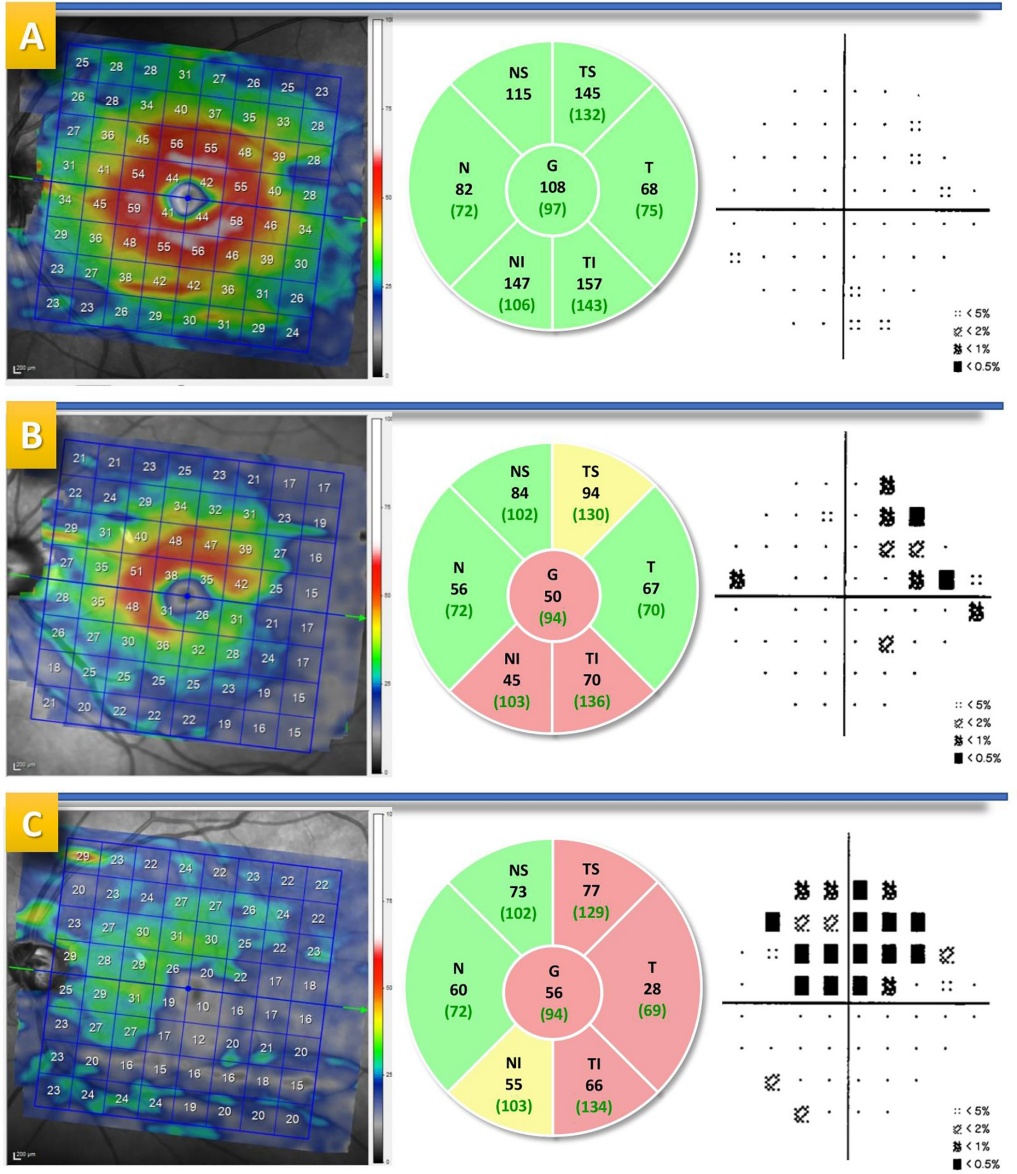
Three patients with their visual fields and OCT. Left images represent the ganglion cell layer (GCL), the centre images show peripapillary retinal nerve fiber layer (pRNFL) analysis, and the right images represent the visual fields (VF) deviation map. A: Control eye of a 40-year-old woman with non-altered GCL and pRNFL analysis as well as normal VF (A pattern); B: 72-year-old man with early glaucoma diagnosed with temporal-inferior damage of GCL associated with temporal inferior (TI) and average impairment as well as temporal superior (TS) borderline damage and VF with F pattern (nasal quadrant) in superior hemisphere; C: 78-year-old woman with advanced glaucoma and severe inferior damage of GCL analysis accompanied by temporal (TS, T, TI) and average injury of pRNFL measurement and VF with H pattern (expansive arcuate) in superior hemisphere.

A single, well-trained ophthalmologist (ALE) performed all the OCT examinations. The retinal thickness was measured with the Spectralis OCT (Heidelberg Engineering, Heidelberg, Germany) using the images obtained by PPA scans. Using this protocol, the OCT instrument automatically delineates a line joining the centre of the fovea and the centre of the optic disc as a reference line. Thereafter, 61 line scans (1024 A scans/line) parallel to the central reference line were recorded. The quality of the scans was noted by a colour scale at the bottom of the scanned images. Only placement in the green range was considered to be a good quality scan for inclusion in this study. A masked investigator (SF) examined all of the images of each eye to determine whether there were any segmentations or centred errors in the images. The average retinal layers measurement of each 8x8 (3°x 3°) sector (64 sectors) was determined. Since glaucoma first damages the centre of the macula [23], only 4x4 central grids were analysed to expedite the study. These 16 sectors were numbered as shown in Fig 1, with temporal (T), nasal (N), superior (S) and inferior (I) added to ease the understanding. A superior cluster included numbered 1–8 sectors, whereas the inferior cluster included 9–16 sectors. In addition, the PPA algorithm generated the total retinal thickness asymmetry (RTA) analysis, which is an asymmetry test that automatically compares superior and inferior total retinal thicknesses. Moreover, the GCL deviation map is a colour scale representation of the topographic damage of the GCL, on which number values are assigned colours from white to blue to represent maximum or minimum GCL thicknesses, respectively. Normal values appear as red and uncoloured areas indicate an extremely lower GCL thickness. Asymmetry of the GCL and RTA was subjectively examined by a masked and experienced investigator (RF) who was blinded to glaucoma status (Fig 2).

To stablish a new test of asymmetry between the GCL sectors, hemisphere comparison of clusters was assessed (TS1 vs TI13, TS2 vs TI14, TS5 vs TI9, TS6 vs TI10 and NS7 vs NI11).

Circumpapillary pRNFL analysis consists of a cross-sectional imaging of the peripapillary area, and was performed using Spectralis OCT, which simultaneously captures infrared fundus and SD-OCT images at 40,000 A-scans per second. A real-time eye-tracking system measures eye movements and provides feedback to the scanning mechanism to stabilize the retinal position of the B-scan. The instrument uses 1024 A-scan points from a 3.45 mm circle centred on the optic disc. The examiner is required to manually place the scan around the optic disc. To represent data, a colour code map is generated: a green sector represents the range above the 5th percentile of the distribution in normal eyes, and is considered as “within normal limits”. A yellow sector represents the range below the 5th percentile but above the 1st percentile, and is considered as “borderline”. A red sector represents the range below the 1st percentile and is considered as “outside normal limits”. Abnormality of temporal superior (TS) and temporal inferior (TI) pRNFL was registered.

### Visual field

**Fig 3:**
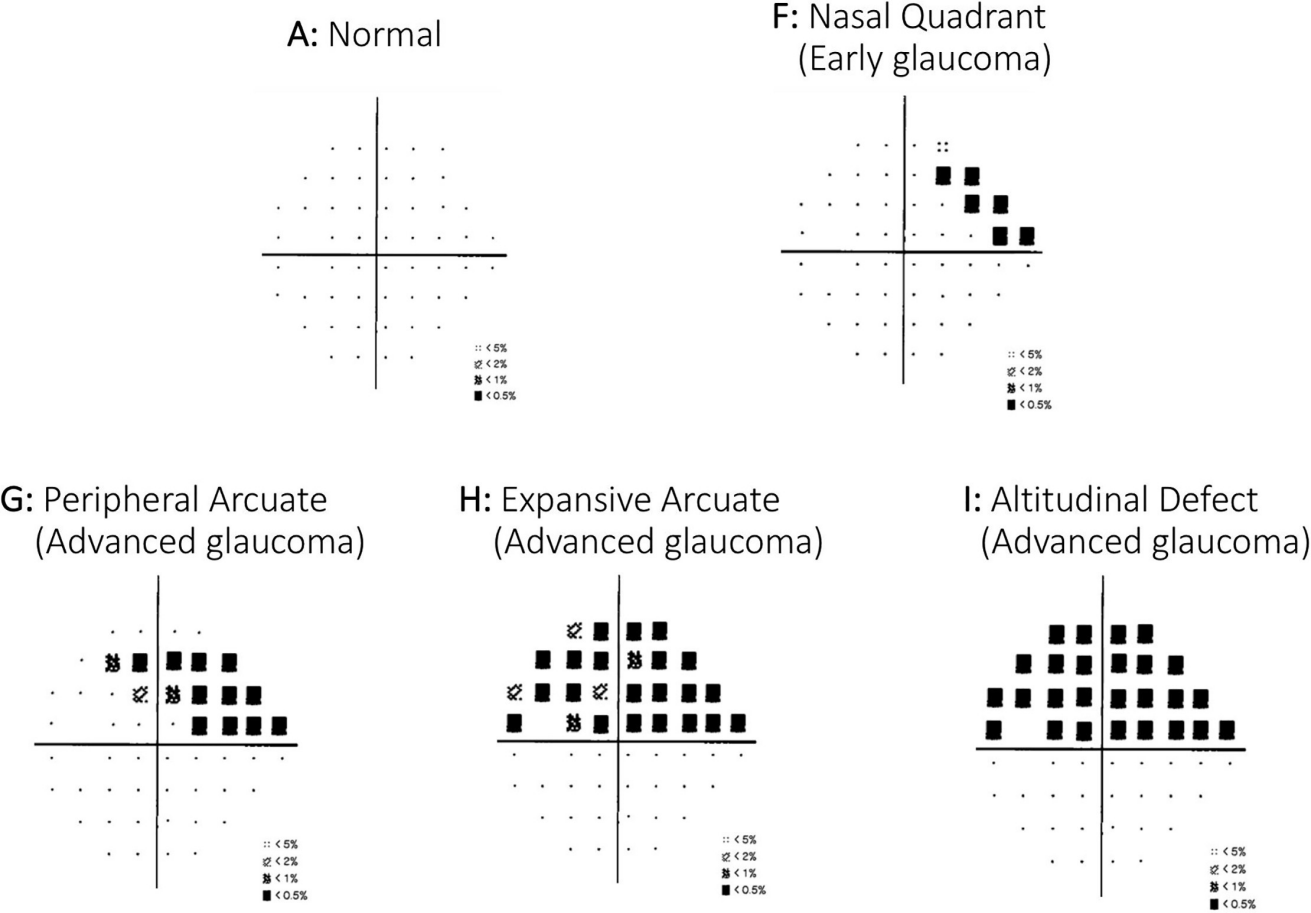
Grade of VF impairment used in this study. A: Normal VF (no defects anywhere on the field), F: nasal quadrant (any defect within the nasal zone), G: peripheral arcuate (a combination of defects that must cross the midline and does not include the central two points), H: expansive arcuate (a combination of defects that must cross the midline, and includes adjoining defects in the central scotoma region, paracentral region, and temporal or nasal quadrant region), and I: altitudinal defect (all of the points above or below the horizontal meridian have a defect of <0.5%).

The Swedish interactive threshold algorithm standard strategy, program 24–2 of the Humphrey Field Analyser (Carl Zeiss Meditec, Jena, Germany), was used for VF testing of each eye. Reliability criteria were fixation losses of 20% or less, false positive results of 15% or less, and false-negative results of 33% or less. The VF was classified as normal when there was a mean deviation or a pattern standard deviation within the 95th percentile, a normal glaucoma hemifield test, and the absence of a cluster of three or more non-edge points on the pattern deviation plot with a probability of occurring in <5% of the normal population, with one of these points having the probability of occurring in <1% of the normal population. The Scheie VF grading system was used to classify and grade VF impairment as showed in Fig 3 [24]. This system must also detect small changes over time, serving as an accurate proxy of glaucoma progression [23]. Classification was as follows: A: Normal VF (no defects anywhere on the field), B: Central scotoma (defect within the central 5 degrees), C: Paracentral scotoma (defects within the paracentral region that do not cross the midline), D: paracentral crescent (defects in the paracentral region that do cross the midline), E: temporal quadrant (any defect within the temporal zone), F: nasal quadrant (any defect within the nasal zone), G: peripheral arcuate (a combination of defects that must cross the midline and does not include the central two points), H: expansive arcuate (a combination of defects that must cross the midline, and includes adjoining defects in the central scotoma region, paracentral region, and temporal or nasal quadrant region), and I: altitudinal defect (all of the points above or below the horizontal meridian have a defect of <0.5%). We classified superior and inferior halves of VF and graded them from A to I. Essentially, we used F damage of VF as early glaucoma, and G, H and I as advanced glaucoma. If no damage was found in either the superior or inferior halves of the VF (A pattern), the eye was considered a control eye.

### Statistical analysis

A 1-sample Kolmogorov-Smirnov test was used to verify the normality of the data distribution. Paired Student t-test was used to compare different sectors of the GCL and pRNFL between eyes with VF damage and control eyes. The GCL map deviation and pRNFL TS and TI affected-quadrants differences were assessed with Fisher’s test. Pearson correlation test was used to analyse the relationship between the GCL sectors and damage of the VF. A receiver operating characteristic (ROC) curve was used to assess the discrimination value of the OCT analyses. We employed area under receiver operating characteristic curves (AUCs) to assess the ability of the GCL and pRNFL thicknesses in order to discriminate glaucoma patients from healthy controls. [25]. All statistical analyses were performed using IBM SPSS Statistics V.20.0 (International Business Machine Corporation, Armonk, NY, USA). The level of statistical significance was set at P < 0.05.

## Results

Overall, 318 eyes of 161 patients were included in the study. Only one eye of four patients was used due to low reliability VF indexes of the fellow eye. The mean age of the patients was 64.2 ± 12.9 years (age range: 18–88 years), and 71 patients were female (44.1%), as it is showed in **Table 1**. First, we classified the damage of each hemisphere (superior and inferior) of the VF using the Scheie grading system [24]. Hence, regarding inferior VF damage, 202 (63.5%) eyes presented a normal inferior hemisphere VF (A pattern), 49 (15.4%) eyes had an F pattern of VF (early glaucoma), and 116 (36.5%) eyes had an F or worse damage of VF (G, H or I patterns; early and advanced glaucoma). Similarly, concerning superior VF damage, 211 (66.4%) eyes presented a normal superior hemisphere VF 33 (10.4%) eyes had an F pattern of VF, and 107 (33.6%) eyes had an F or worse damage of VF. Using this grading system, we separately compared eyes with A pattern (control eyes) with early glaucoma and early with advanced glaucoma in each hemisphere. The results are shown in **Table 2**. All the sectors of the GCL were significantly thinner in eyes with any VF damage (F to I damage) than in control eyes, except NS4 and NI12 sectors, which are nasally located. Similarly, subjective asymmetry of the GCL map and pRNFL (average and temporal superior or inferior analyses) also showed significant differences between control and glaucoma eyes. However, asymmetry RTA of PPA did not show a significant difference between these eyes. Regarding the comparison between early glaucoma and control eyes, eyes with early glaucoma showed lower values of each measurement, but these differences were only significant for subjective asymmetry of the GCL in cases of inferior VF damage analysis and pRNFL TI in cases of superior VF damage.

**Table 1:** Demographic and clinical participant´s characteristics.

	Overall (N = 161)
Age (years)	64.2 (12.9)
Female eyes (%)	71 (44.1)
Spherical equivalent (Diopters)	0.77 (2.25)
IOP	14.6 (2.3)

Demographic and clinical characteristics of the participants such as age, percentage of female eyes, spherical equivalent and IOP.

**Table 2:** Comparison between control eyes, early glaucoma and advanced glaucoma.

**A** Superior GCL vs inferior VF	A inferior VF (N = 202)	F inferior VF (N = 49)	**P**	F-I inferior VF (N = 116)	**P**
GCL TS1	39.9 (7.1)	38.3 (7.8)	0.149	35.2 (9.6)	<0.001*
GCL TS2	47.3 (7.7)	45.5 (8.3)	0.154	42.7 (10.1)	<0.001*
GCL NS3	46.6 (6.9)	45.6 (7.8)	0.374	43.5 (9.1)	0.001*
GCL NS4	41.5 (11.6)	39.1 (6.2)	0.445	37.5 (7.2)	0.082
GCL TS5	42.3 (8.9)	39.7 (9.9)	0.082	36.8 (11.6)	<0.001*
GCL TS6	33.8 (7.6)	31.9 (8.2)	0.113	30.3 (8.8)	0.001*
GCL NS7	35.6 (7.6)	33.7 (8.2)	0.121	32.7 (8.8)	0.006*
GCL NS8	49.7 (7.2)	48.2 (8.3)	0.184	46.6 (9.3)	0.002*
Superior GC	336.8 (55.4)	321.9 (58.4)	0.098	305.3 (68.9)	<0.001*
RTA	61 (30.1)	18 (36.7)	0.454	34 (29.3)	0.415
GCL map (%)	44 (21.8)	38 (77.5)	0.039*	72 (62.0)	0.001*
RNFL	87.7 (15.4)	84.1 (19.9)	0.181	77.7 (22.6)	<0.001*
RNFL TS (%)	52 (25.7)	22 (44.9)	0.236	68 (58.6)	0.004*
**B** Inferior GCL vs superior VF	A superior VF (N = 211)	F superior VF (N = 33)	**P**	F-I superior VF (N = 107)	**P**
GCL TI9	45.5 (10.2)	44.8 (10.4)	0.277	38.9 (12.9)	<0.001*
GCL TI10	34.5 (8.4)	34.8 (7.9)	0.898	31.2 (8.8)	0.008*
GCL NI11	33.8 (8.0)	34.2 (7.5)	0.764	30.1 (8.3)	0.046*
GCL NI12	49.8 (8.6)	50.2 (7.9)	0.419	46.4 (10.2)	0.066
GCL TI13	39.3 (7.6)	38.6 (7.9)	0.300	35.7 (9.5)	<0.001*
GCL TI14	46.2 (8.7)	45.9 (8.7)	0.820	42.6 (10.3)	<0.001*
GCL NI15	46.2 (8.3)	46.1 (7.9)	0.395	43.2 (9.7)	0.011*
GCL NI16	40.4 (6.7)	40.3 (6.6)	0.217	38.5 (7.9)	0.043*
Inferior GC	335.8 (60.2)	334.8 (57.1)	0.938	318.3 (112.8)	0.025*
RTA	130 (61.6)	21 (63.6)	0.665	67 (62.6)	0.441
GCL map (%)	55 (26.1)	10 (30.3)	0.527	61 (57.0)	0.020*
RNFL	88.1 (17.2)	87.7 (15.5)	0.433	77.1 (22.7)	<0.001*
RNFL TI (%)	50 (23.6)	17 (51.5)	0.006*	52 (48.6)	<0.001*

**A. (Right)** Comparison of superior Ganglion Cell Layer’s (GCL) values, total retinal thickness asymmetry (RTA) analysis and Retinal Nerve Fiber Layer (RNFL) with inferior visual field degrees (VF) between control eyes (CE) and impaired VF eyes. **(Left)** Same comparison of superior GCL, RTA and RNFL with inferior VF between CE and early glaucoma VF eyes (F pattern VF defect). **B (Right)** Comparison of inferior GCL values, RTA and RNFL with superior VF degrees between CE and impaired VF eyes. **(Left)** Same comparison of inferior GCL, RTA and RNFL with superior VF between CE and early glaucoma VF eyes (F pattern VF defect). Data for quantitative variables are shown as the mean (standard deviation). GCL map, RTA and RNFL temporal superior (TS) or inferior (TI) affected-quadrants differences were assessed with Fisher’s test. The rest of analysis was performed using paired Student’s t-test for dependent samples.

* p value is <0.05.

**Table 3** shows the correlation between each sector of the GCL and damage of VF graded A to I. Each sector of GCL showed a significant correlation (p≤0.045) with VF damage. Subjective GCL map analysis and pRNFL (average, TS and TI) showed a significant correlation (p≤0.005). However, asymmetry RTA test of PPA did not show a significant difference. Higher R values of correlation were observed in temporal sectors of GCL (1, 5, 9 and 13, R = -0.33, -0.30, -0.32 and -0.30, respectively) as well as in average pRNFL (R<-0.30).

**Table 3:** Correlation between each sector of GCL and damage of VF graded A to I.

A	Inferior VF pattern		B	Superior VF pattern	
	R	P		R	P
GCL TS1	-0.334	<0.001	GCL TI9	-0.319	<0.001
RNFL	-0.311	<0.001	RNFL	-0.303	<0.001
GCL TS2	-0.306	<0.001	GCL TI13	-0.300	<0.001
GCL TS5	-0.304	0.004	GCL TI14	-0.276	<0.001
Superior GCL	-0.291	<0.001	GCL NI15	-0.224	<0.001
GCL NS3	-0.243	<0.001	GCL TI10	-0.207	<0.001
GCL TS6	-0.230	<0.001	Inferior GCL	-0.196	0.001
GCL NS8	-0.226	<0.001	GCL NI16	-0.194	0.001
GCL NS7	-0.206	<0.001	GCL NI12	-0.174	0.003
GCL NS4	-0.117	0.045	GCL NI11	-0.171	0.003
RTA	0.045	0.437	RTA	0.103	0.075
RNFL TS	0.235	0.002	GCL map	0.220	0.005
GCL map	0.265	0.001	RNFL TI	0.415	<0.001

Pearson correlation of inferior VF patterns (A to I) with different sectors of ganglion cell layer (GCL) (1–8: superior sectors and 9–16: inferior sectors), total superior and inferior GCL, GCL map deviation, total retinal thickness asymmetry (RTA) analysis and average retinal nerve fiber layer (RNFL), as well as temporal superior (TS) and inferior (TI) RNFL. **A**: comparison of superior GCL and RNFL with inferior VF damage. **B**: comparison of inferior GCL and RNFL with superior VF damage.

**Table 4** shows AUC of ROC analysis with 95% confidence limits for sensitivity and specificity of different OCT measurements. The highest AUC values to diagnose both early and advanced glaucoma correspond to average pRNFL analysis (AUC>0.823, p<0.040). The GCL map deviation analysis also showed higher values than the RTA test. The TS1 vs TI13 and TS2 vs TI14 clusters of sectors of the GCL showed a slight improvement in AUC compared with the GCL map and pRNFL in superior early glaucoma detection (AUC >0.867, p<0.020).

**Table 4:** Area Under the curve (AUC) of different OCT measurements.

**A VF INFERIOR**	**AUC F vs CE**	**P**	**A VF INFERIOR**	**AUC F-I vs CE**	**P**
RNFL	0.832	0.040*	RNFL	0.823	0.022*
GCL map deviation	0.786	0.051	GCL map deviation	0.806	0.030*
Difference TS5-TI9 GCL	0.767	0.069	Difference 5–9 GCL	0.755	0.071
Difference TS1-TI13 GCL	0.705	0.162	Difference TS1-TI13 GCL	0.716	0.125
Difference NS7-NI11 GCL	0.676	0.229	Difference TS2-TI14 GCL	0.706	0.143
Difference TS2-TI14 GCL	0.648	0.313	Difference NS7-NI11 GCL	0.671	0.225
RTA	0.643	0.329	RTA	0.645	0.303
Difference TS6-TI10 GCL	0.576	0.303	RNFL asymmetry	0.642	0.314
RNFL asymmetry	0.590	0.537	Difference TS6-TI10 GCL	0.606	0.450
**B VF SUPERIOR**	**AUC F vs CE**	**P**	**B VF SUPERIOR**	**AUC F-I vs CE**	**P**
Difference TS1-TI13 GCL	0.883	0.015*	RNFL asymmetry	0.912	0.006*
Difference TS2-TI14 GCL	0.867	0.020*	RNFL	0.888	0.010*
RNFL	0.825	0.040*	Difference 5–9 GCL	0.865	0.015*
Difference 5–9 GCL	0.767	0.092	Difference TS1-TI13 GCL	0.853	0.019*
GCL map deviation	0.708	0.188	Difference TS2-TI14 GCL	0.824	0.031*
RNFL asymmetry	0.633	0.399	GCL map deviation	0.824	0.031*
RTA	0.608	0.493	Difference NS7-NI11 GCL	0.576	0.611
Difference TS6-TI10 GCL	0.517	0.916	Difference TS6-TI10 GCL	0.518	0.906
Difference NS7-NI11 GCL	0.508	0.958	RTA	0.512	0.938

Area under the curve (AUC) of the receiver operating characteristic curve (ROC) analysis with 95% confidence limits for sensitivity and specificity of the difference between distinct sectors of ganglion cell layer (GCL), GCL deviation map, total retinal thickness asymmetry (RTA) analysis, retinal nerve fiber layer (RNFL) and RNFL asymmetry in the diagnosis of early glaucoma (F type of VF pattern) from control eyes (**left**) as well as in the diagnosis of all types of glaucoma (F-I VF patterns) (**right**). **Table 3 A** assessed inferior hemispheres of VF and **Table 3B** assessed the superior hemispheres.

* p value is <0.05.

## Discussion

Our results showed that the values of all 16 sectors of the GCL in the centre of the macula measured with PPA are correlated with the severity of the damage of the corresponding VF, except for one nasally located sector, and that their values are different between control and damaged VF-eyes. Nevertheless, the principal objective of this study was to elaborate an asymmetry analysis of the thicknesses of the different clusters of the GC to improve previous analysis, such as the pRNFL or GCL map deviation. We found that the best analysis to diagnose both early and advanced glaucoma generally corresponds to the average pRNFL analysis. However, asymmetry tests comparing 1 vs 13 and 2 vs 14 clusters of sectors of the GCL provides better values of sensitivity and specificity than the pRNFL in early glaucoma if the VF defect starts in the superior hemisphere.

This purpose was settled due to the developing concern on the importance of analysing the macula in glaucoma diagnosis and management [26]. Glaucomatous damage to the macula might occur early in the disease and can be minimized with standard VF tests [27], or even ignored [28]. Moreover, the GCL thickness analysis shows less variability and it is more useful in glaucoma diagnosis than the conventional pRNFL and optic disc parameters [29, 30]. SD OCT technology has allowed faster and better acquisition of optic nerve and retinal images than previous time-domain OCT technology [31], thereby permitting the differentiation of each retinal layer [32]. These improvements allow for the measurement of the GCL, which might be the first cells damaged in glaucoma and, therefore, the first target to diagnose early glaucoma [8–11, 14,15].

Depending on the OCT device, the strategy to assess the GCL could be diverse. The Cirrus HD-OCT (Carl Zeiss Meditec) measures the GCL together with the IPL (GCIPL), whereas the RTVue OCT (Optovue, Inc., Fremont, CA) analyses the entire ganglion cell complex (RNFL, GCL, and IPL). Most of the GCL analyses in literature have been performed using Cirrus HD-OCT [12–15, 23]. However, PPA is a relatively new software of the Spectralis OCT that allows for measurement of the GCL individually as well as all the retinal layers. This new software has been tested in previous reports [20, 33].

In this report, we focus on the analysis of PPA evaluating the asymmetry of the GCL and pRNFL, since in eyes with glaucoma, the inter-hemispheric anatomic symmetry is not preserved, which is different from healthy eyes. Thus, significant asymmetry when comparing healthy eye with eyes with glaucomatous might indicate glaucomatous damage. These asymmetry tests have been performed with Cirrus HD-OCT and have shown reliable sensitivity and specificity values (AUC>0.913) [21, 34]. Regarding PPA, this test provides an automatic asymmetry test that compares superior and inferior hemifields of the total retina thickness (RTA) of the macula. This is shown to the ophthalmologist in white to black squares in order to facilitate understanding. Um et al.[35] reported that this test has a higher sensitivity than the pRNFL in diagnosing early glaucoma, although they found no statistical differences between RTA and pRNFL when diagnosing advanced glaucoma. These authors performed five zones in the RTA map to improve the automatic report [35]. However, both Sullivan-Mee et al. [13] and Seo et al. [20] found that the pRNFL provides higher values of AUC than RTA. The authors analysed the AUC of the average, inferior and superior pRNFL as well as RTA, and found a greater AUC for the average pRNFL (0.937) and lower values for RTA (0.872) [13]. Similarly, Seo et al. found higher values of sensitivity and specificity of the pRNFL compared to RTA [20]. Interestingly, these authors performed an analysis of two to four consecutive black cells in RTA providing an AUC of 0.958. On the other hand, Yamada et al. [36] developed an interesting formula that compared superior and inferior hemifields of three different macular layers: RNFL, CGL and GCL plus IPL. The better results corresponded to GCL analysis. Hence, we believe that the GCL asymmetry test might be useful in clinical practice, and might be better than the pRNFL and RTA. First, we proved that each sector of the GCL was correlated with the severity of VF and that there were differences between control and glaucomatous eyes, which was not previously reported using Spectralis OCT and this PPA. Second, as Spectralis OCT did not provide any automated asymmetry test using the GCL, we investigated if some clusters of paired GCL sectors could be used more easily in early glaucoma detection than Yamada’s proposed formula. We found that, similar to the studies by Sullivan-Mee et al. [13] and Seo et al. [20], the AUC was greater for the pRNFL than RTA. Nevertheless, the new proposed paired-GCL test (specifically TS1 vs TI13 and TS2 vs TI14 clusters) presented similar values for detecting glaucoma and higher values of AUC for early glaucoma detection than the pRNFL but only in cases where the damage started superiorly. If the damage of the VF started inferiorly, this paired-GCL test might be accurate but the pRNFL presented higher values of AUC. Thus, in daily clinical practice, the pRNFL might remain as the best analysis to detect both early and advanced glaucoma, but the GCL asymmetry test could be used in cases of false positive pRNFL or in case of doubt.

Likewise, the GCL analysis using OCT Spectralis provides a GCL map deviation. Although a normative database is not currently available for this analysis, asymmetry tests might be subjectively performed and could be easily used in clinical practice. One of the aims of this study was to determine if a masked and experienced investigator could be able to use this map to diagnose glaucomatous eyes. We found a significant correlation of this test with VF damage and its ability to differentiate glaucomatous eyes from control eyes. Moreover, we discovered that although the values of AUC were lower than the pRNFL in detecting early glaucoma cases (AUC <0.786, p>0.188), in cases of glaucoma, the AUC was comparable to the pRNFL (p>0.806, p<0.031). Thus, this map might be used in clinical practice with interesting acceptance and was reported using Cirrus HD-OCT [14, 15].

Several limitations are present in this study. First, we assumed the control eyes to be eyes with a normal visual field, but these eyes might present anatomical impairment in OCT not yet detected in VF. However, these anatomical changes might be produced up to 8 years before the changes in the VF [37], and thus we might assume this possible mistake in order to perform these transversal studies. Second, we focused on the GCL and did not use other measurements given in PPA, such as macular RNFL and macular IPL analyses. However, Yamada et al. [36] previously used these measurements with PPA and concluded that the single GCL-asymmetry test was the most accurate in detecting early glaucoma, and our aim was to propose an easy test for clinical practice. Third, we have used only the central 16 sectors of the GCL. This limitation was because the location of the large superior and inferior vessels, which are not necessarily symmetrical across the hemispheres of the retina, thereby affecting the symmetry of upper and lower GCL thicknesses [38]. Other limitations included an ethnically homogeneous (all were Caucasian) sample and the relatively small sample size.

In conclusion, our findings showed that with the new Spectralis PPA, GCL segmentation might be used as a complementary and reliable marker in glaucoma assessment. Comparison of the paired GCL sectors should be used carefully; however, it might reveal early cases of glaucoma. Similarly, attention should be drawn to the GCL map, as this subjective analysis by an experienced ophthalmologist might be as useful as the GCL and pRNFL tests. However, our findings proved that the pRNFL appeared to be the most reliable test in detecting both early and advanced glaucoma.

## Supporting information

S1 Data Raw data used in the study. This table of data represents the original values of the patients' charachteristics unprocessed.
(RAR)
